# Sequencing the genome of *Lactobacillus iners* strain SPIN 2503V10-D for improved quality and completeness

**DOI:** 10.1128/mra.01477-25

**Published:** 2026-05-29

**Authors:** Treena Rica D. Teh, Nicole R. Jimenez, Melissa M. Herbst-Kralovetz

**Affiliations:** 1 Department of Basic Medical Sciences, The University of Arizona College of Medicine-Phoenix 42283, Phoenix, Arizona, USA; 2 College of Medicine, University of the Philippines Manila 54733, Manila, Philippines; 3 Department of Obstetrics and Gynecology, The University of Arizona College of Medicine-Phoenix 42283, Phoenix, Arizona, USA; University of Wisconsin-Madison, Madison, Wisconsin, USA

**Keywords:** *Lactobacillus*, *Lactobacillus iners*, vaginal microbiome, genome

## Abstract

We sequenced and annotated the genome of *Lactobacillus iners* SPIN 2503V10-D, a human vaginal isolate previously sequenced as a reference genome for the Human Microbiome Project, to refine its existing genomic data.

## ANNOUNCEMENT

*Lactobacillus iners* is among the most prevalent species in the cervicovaginal microbiome (1, 2). Although *Lactobacillus*-dominated microbiomes are generally associated with gynecologic health (3, 4), *L. iners* is paradoxically associated with both health and disease across observational studies (5–13). Its conflicting role in gynecologic health is postulated to be partially attributable to strain-level variation (14, 15).

The genome of *L. iners* SPIN 2503V10-D, a human vaginal isolate, was sequenced as a reference genome for the Human Microbiome Project (16). This assembly was noted to have many frameshifted proteins and has been suppressed from the RefSeq database. To refine existing genomic data on this strain, we sequenced its genome utilizing hybrid long- and short-read approaches.

*L. iners* SPIN 2503V10-D was obtained from Biodefense and Emerging Infections Research Resources Repository (BEI Resources) and grown anaerobically on tryptic soy agar with 5% sheep’s blood at 37°C for 48 hours. Colonies were scraped off a pure culture, washed twice by suspension in 1 mL sterile Dulbecco’s phosphate-buffered saline followed by centrifugation at 6,000 × *g*, resuspended in 500 μL DNA/RNA Shield (Zymo Research, cat. R1100-50), and sent to Plasmidsaurus, Inc., for genomic DNA extraction, sequencing, and assembly, described as follows. Additional technical documentation can be found at https://plasmidsaurus.com/technical-documentation/genome; however, some information is proprietary and was not shared with the research team. DNA was extracted using Zymo Quick-DNA Miniprep Plus Kit (cat. D4069). The genome was sequenced using Oxford Nanopore Technology (ONT) R10.4.1 flow cells and Illumina NovaSeq X Plus. Basecalling was done using Dorado v4.3 Super-Accurate Basecalling with default Q10 quality filtering (17). Multiple subsampled read sets were generated using Autocycler v0.5.2 with optimal coverage for each subsample (18). Assembly was performed using Autocycler with three different assemblers: Flye v2.9.6 with parameters optimized for high-quality ONT reads (19), Hifiasm-0.25.0-r726 for generating high-quality assemblies from long reads (20–22), and Plassembler v1.8.0 for detecting plasmids (23). The ONT assembly was polished with Illumina reads using Polypolish v0.6.0 (24, 25). The species was identified using Sourmash v4.6.1 (26, 27) against Genome Taxonomy Database Release 226 (28). Annotation and subsystem analysis were performed using default parameters of the Comprehensive Genome Analysis (CGA) service of the Bacterial and Viral Bioinformatics Resource Center (BV-BRC) (29) and submitted to GenBank utilizing NCBI PGAP (30). PATRIC provided the reference and representative genomes and included them in the phylogenetic analysis that was part of the CGA utilizing Mash/MinHash v2.3 (31, 32). Other software utilized in the BV-BRC pipeline included Racon v1.4.3 (33) and Circos v0.69-5 (34).

**Fig 1:**
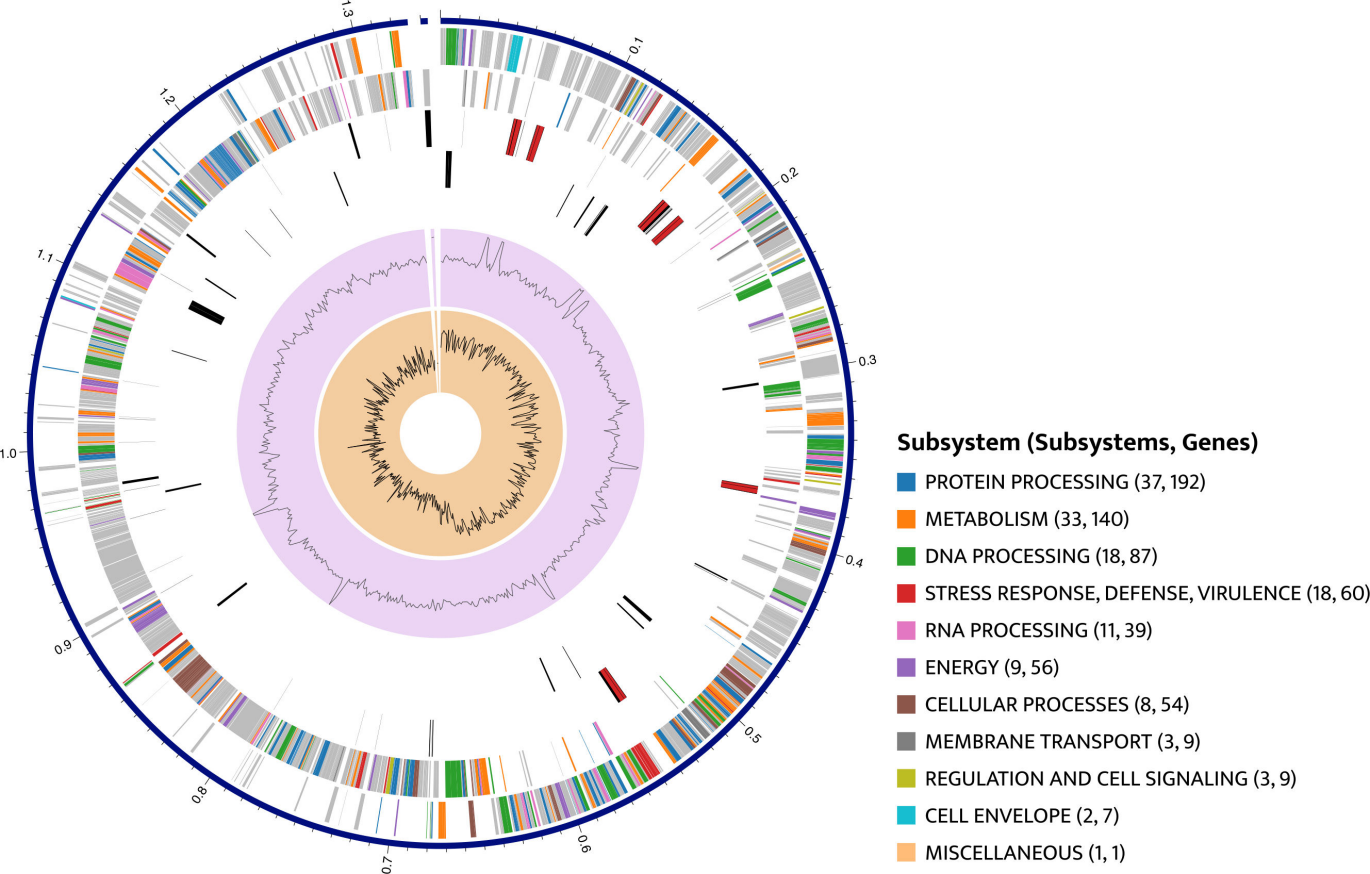
Circular graphical representation of the distribution of genome annotations for *L. iners* SPIN 2503V10-D. Outer to inner rings represent contigs, protein-coding sequences (CDSs) on the forward strand, CDSs on the reverse strand, RNA genes, CDSs with homology to known antimicrobial resistance genes, CDSs with homology to known virulence factors, GC content, and GC skew. Colors of CDSs on the forward and reverse strands represent the subsystem to which these genes belong.

The assembled genome comprised two contigs, without plasmids, a total length of 1,332,666 bp, and a GC content of 33.15%. Our new assembly exhibits higher contiguity with an N50 of 1,328,851 bp, compared to the previous assembly’s N50 of 93,034 bp. Seventy tRNA genes, 18 rRNA genes, and 1,232 protein-coding sequences (CDSs) were annotated, of which 1,002 had functional assignments. PATRIC annotated 41 more CDSs compared to PGAP, likely due to PGAP’s more conservative gene prediction process (30, 32). Protein processing was identified as the largest functional group (192 genes). In contrast to the larger contig, the smaller contig contained only one hypothetical protein and one repeat region. Table 1 and Fig. 1 summarize genome characteristics and annotations.

**TABLE 1:** Summary of genome characteristics for *L. iners* SPIN 2503V10-D

Genome characteristics	This study		Previous genome assembly	
	RefSeq	BV-BRC	RefSeq	GenBank
Raw reads	395,599 (ONT) 16,437,177 (Illumina)		312,929	
Genome size (bp)	1,332,666		1,283,897	
Number of contigs	2		31	
Contig N50 (bp)	1,328,851		93,034	
Contig L50	1		6	
GC content (%)	33.15		32.57	
Genome coverage	101.5× (assembly coverage) 3,724.0× (raw read coverage)		52×	
Annotation details	NCBI PGAP 8 December 2025	PATRIC 9 September 2025	NCBI PGAP 11 July 2021	Annotation submitted by J. Craig Venter Institute 7 June 2013
Number of tRNAs	70	70	48	46
Number of rRNAs	18	18	3	1
Number of protein-coding sequences	1,191	1,232	1,095	1,273

## Data Availability

The whole-genome sequences of *L. iners* SPIN2503V10-D were deposited in the Sequence Read Archive (SRA) with accession numbers SRR36298223 (Illumina) and SRR36298224 (ONT), under BioProject PRJNA1373291. The BioSample identification number is SAMN00115035. The NCBI PGAP annotation can be found at GenBank JBSSWT000000000.1. The polished assembly and additional information on genome assembly, annotation, and subsystem analysis are available at BV-BRC (https://www.bv-brc.org/workspace/jimeneznr@patricbrc.org/Lactobacillus%20iners%20SPIN%20250310-D), accessible upon free account creation. Additional technical details on Plasmidsaurus, Inc.’s sequencing and assembly pipeline are available at https://plasmidsaurus.com/technical-documentation/genome.
